# Directional cloning of DNA fragments using deoxyinosine-containing oligonucleotides and endonuclease V

**DOI:** 10.1186/1472-6750-13-81

**Published:** 2013-10-04

**Authors:** Tobias Baumann, Katja M Arndt, Kristian M Müller

**Affiliations:** 1 Institute of Biochemistry and Biology, University of Potsdam, Karl-Liebknecht-Str. 24-25, Potsdam 14476, Germany; 2 Cellular and Molecular Biotechnology, Faculty of Technology, Bielefeld University, Room UHG E2-143 Universitätsstr. 25, Bielefeld 33615, Germany

**Keywords:** Cohesive ends, DNA cleavage, Genetic vectors, Modified primers, Molecular methods, Polymerase chain reaction, Recombinant *Escherichia coli*, Restriction enzymes

## Abstract

**Background:**

DNA fragments carrying internal recognition sites for the restriction endonucleases intended for cloning into a target plasmid pose a challenge for conventional cloning.

**Results:**

A method for directional insertion of DNA fragments into plasmid vectors has been developed. The target sequence is amplified from a template DNA sample by PCR using two oligonucleotides each containing a single deoxyinosine base at the third position from the 5′ end. Treatment of such PCR products with endonuclease V generates 3′ protruding ends suitable for ligation with vector fragments created by conventional restriction endonuclease reactions.

**Conclusions:**

The developed approach generates terminal cohesive ends without the use of Type II restriction endonucleases, and is thus independent from the DNA sequence. Due to PCR amplification, minimal amounts of template DNA are required. Using the robust *Taq* enzyme or a proofreading *Pfu* DNA polymerase mutant, the method is applicable to a broad range of insert sequences. Appropriate primer design enables direct incorporation of terminal DNA sequence modifications such as tag addition, insertions, deletions and mutations into the cloning strategy. Further, the restriction sites of the target plasmid can be either retained or removed.

## Background

With hundreds of enzymes commercially available today [1], restriction endonuclease treatment of insert and plasmid vector DNA followed by ligation and transformation into competent *E. coli* strains presents the standard cloning method in molecular biology. Given the advances in structural biology and the advent of synthetic biology, a strong demand exists to transfer and rearrange a large variety of DNA fragments from different genetic sources in a directed manner. A diverse catalogue of plasmid vectors is at hand for propagation in pro- and eukaryotic cells, enabling heterologous protein expression in various host organisms. Frequently, suitable pairs of Type II restriction enzymes with unique recognition sites in the vector and insert DNA fragments can be found, especially since the latter are easily produced via PCR. In such a case, the PCR primers contain add-on tails composed of the restriction endonuclease recognition sequence and additional nucleotides which ensure efficient enzymatic processing [2]. Especially with an increasing size of the insert, however, the chance rises that it contains a recognition site of the desired restriction enzymes. Statistically, the 6 bp recognition sequence of a Type II restriction enzyme such as XbaI would occur once in every 4^6^ / 2 = 2048 base pairs. The situation gets worse if one aims to insert multiple sequences in dual-expression vectors, as for instance required for co-expression studies in metabolic engineering, structural and synthetic biology [3-6]. These circumstances require purchase and storage of numerous restriction enzymes or the execution of site-directed mutagenesis (including design and synthesis/purchase of mutagenic primers, high-fidelity PCR, transformation and sequencing) [7,8] in order to remove the unwanted recognition sites. Individual buffer and temperature requirements for endonuclease stability and activity [9] further limit the number of cloning options.

To eliminate the problems of conventional cloning, methods avoiding the use of Type II restriction enzymes have been developed. The Gateway cloning system relies on site-specific recombination catalyzed by a proprietary bacteriophage λ protein formulation *in vitro*[10]. Creation of large recombinant DNA molecules can be achieved by the domino method [11] and DNA assembler [12], which are based on homologous recombination *in vivo* by the machinery of *B. subtilis* or *S. cerevisiae*, respectively. The endogenous recombination system of *E. coli* can combine insert and vector molecules upon co-transfection [13,14], which can be facilitated by expression of a homing endonuclease and bacteriophage recombinases [15]. Similarly, a cell lysate which contains a prophage recombination system can be used *in vitro*[16]. PCR-based generation of complete recombinant plasmids, preferably via a proofreading DNA polymerase, can be achieved by several strategies [17-21]. For the highly complex challenge of genome engineering, homing nucleases [22], transcription activator like (TAL) [23] and zinc-finger nucleases [24] can be used.

More similar to the conventional restriction-ligation system, compatible cohesive ends can be generated in alternative ways. Combined with a subsequent ligation reaction that stabilizes the paired ends, exonuclease III [25] or T4 DNA polymerase [26] can be used for their creation. Ligation-independent cloning (LIC) [27] employs longer overhangs resulting in sufficiently stable DNA base pairing for transformation. These can be created by several means, e.g. via T4 DNA polymerase or incomplete PCR [27-29], hybridization of PCR products [30], ribonucleotide-containing primers [31], terminal transferase [32], abasic sites [33], chemical or enzymatic cleavage of phosphorothioated DNA [34,35], or λ exonuclease [36]. Elegant enzyme-based *in vitro* systems have been developed, such as In-Fusion cloning [37], for which the polymerase is known but not the exact composition, as well as the combined isothermal usage of a DNA polymerase, a 5′ exonuclease and DNA ligase, named Gibson assembly cloning [38]. Although several of the described cloning systems with individual advantages and disadvantages are commercially available, many present costly alternatives or demand complex planning.

**Figure 1:**
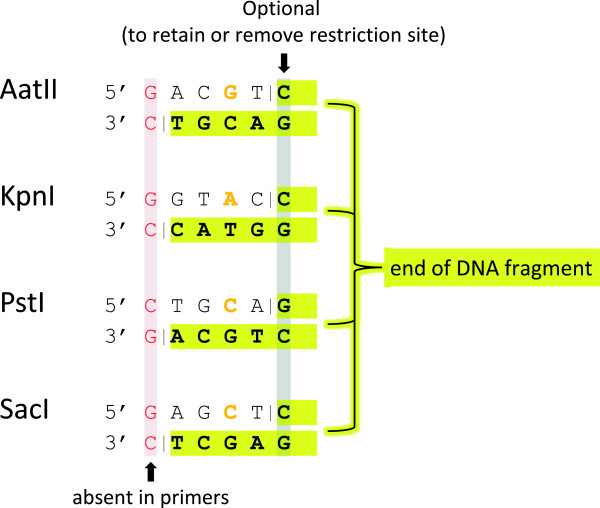
**Cohesive dsDNA ends created in this study.** In order to ligate insert DNA fragments efficiently with a linearized target plasmid vector, both molecules have to carry compatible cohesive ends. For the vector DNA, 5′ recessed ends are created by conventional restriction enzyme treatment. Names and recognition sequences of the enzymes used in this study are listed. For other enzymes, please refer to REBASE [60]. Endonucleolytic cleavage positions are depicted as vertical dashes. Insert DNA fragments with compatible cohesive ends are created by PCR and subsequent endonuclease V treatment (as illustrated in Figure 2). The 5′ ends of the PCR primers and the termini of the corresponding PCR products differ from the shown sequences: They lack the first nucleotide (shown in red) and carry deoxyinosine instead of the residue shown in orange. EndoV treatment of the PCR product results in 5′ recessed ends shown in bold letters with yellow background. If the residue highlighted in grey is omitted from the oligonucleotide design, ligation of the insert fragments with linearized plasmid DNA does not reconstitute the restriction enzyme site.

Smith *et al.* reported a method to create insert fragments with 5′ recessed ends via PCR, utilizing deoxyuracil-containing primers [39]. Treatment of the PCR products with heat or alkaline solution creates 3′ overhangs compatible with those of the vector fragment. In a similar fashion, USER friendly DNA cloning [40] utilizes a commercially available enzyme mix. In contrast to uracil DNA glycosylase (UDG) treatment, this enzyme mix removes the dU residues instead of cleaving the *N*-glycosylic bond. Compatible vectors are generated by treating the plasmid DNA with a nicking and a Type II restriction endonuclease instead of PCR-based amplification. As for other methods, this strategy avoids the risk of introducing polymerase errors into the plasmid backbone. Although cohesive ends can also be generated by using DNA glycosylase-lyase Endo VIII [41] or Endo IV [42] subsequent to UDG, we sought to develop a more straightforward cloning method that requires only one enzyme, no heat- or alkaline treatment and which allows the creation of more 3′ protruding end combinations (see Figure 1 for those created in this study).

Unlike deoxyuracil, the universal base deoxyinosine (dI) can pair with all four canonical DNA nucleobases following a duplex stability series of I:C > I:A > I:T≈I:G [43]. In contrast to several proofreading polymerases [44], *Taq* polymerase can incorporate dITP during primer extension and readily extends dI-containing DNA. These properties allow deoxyinosine usage for the creation of degenerate primers [45,46] as well as for random [47] and sequence saturation mutagenesis [48].

**Figure 2:**
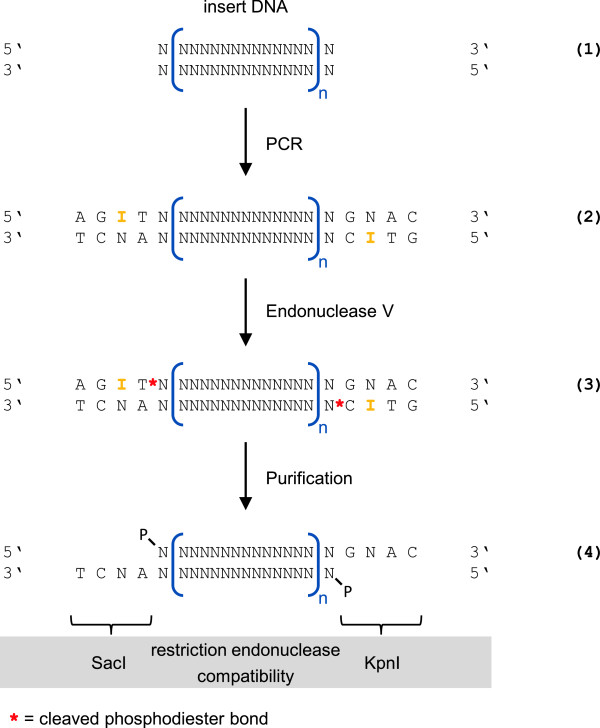
**Scheme for the generation of cohesive ends.** In addition to regions complementary to the insert DNA sequence **(1)**, oligonucleotides are designed with overhangs comprising the 4 bp cohesive part of a restriction site combined with deoxyinosine (dI) at the third position from the 5′ end. Primer annealing and extension during a PCR leads to amplification of the desired target fragment **(2)**, which carries the dI residues (bold, orange) in its termini. The pairing properties of the universal base will generate a sequence distribution at the corresponding site of the opposing strand (indicated as 'N’). Purified PCR products are treated with EndoV, which cleaves the second phosphodiester bond 3′ to dI **(3)**. The target DNA fragment **(4)** is obtained by spin column-based or agarose gel purification, respectively, removing the weakly bound residues of ssDNA. Carrying cohesive ends with 5′ phosphates, the insert fragment is now suitable for ligation to vector DNA fragments created by conventional restriction enzyme treatment (in the depicted case SacI and KpnI).

With deoxyinosine-containing oligonucleotides and endonuclease V (EndoV) readily available from commercial suppliers, a method was developed to create terminal 3′ protruding ends independent of the insert DNA sequence (Figure 2). Appropriate positioning of dI in the primer sequence enables the directional insertion of DNA fragments into plasmid vectors by PCR, endonuclease V treatment and ligation. In order to avoid the introduction of polymerase errors, linearized vectors are created using conventional restriction endonucleases. The applicability of the system is demonstrated by successful cloning of three different coding sequences into several plasmid vectors with efficiencies matching or exceeding those of alternative approaches.

## Results and discussion

### Non-directional ampicillin resistance cassette cloning

In order to establish the proof of concept, the oligonucleotides pUCamp_f and pUCamp_r (Table 1) were designed for the amplification of a 1114 bp region from plasmid pUC18. Insertion of this DNA sequence into a different plasmid vector was expected to confer ampicillin resistance to transformed *E. coli* cells, allowing straightforward detection of recombinant clones. The target sequence includes the P3 promoter [49], the ribosome binding site, the β-lactamase (*bla*) coding region and the terminal TAA stop codon. Both oligonucleotides were designed to form primer-template duplexes with *T*_m_ values of 56–57°C. In order to enable cloning, the 5′ primer ends comprise four additional nucleotides with a deoxyinosine residue at the third position (compare Figure 1). According to previous reports and the crystal structure of the *Thermotoga maritima* (*Tma*) enzyme [50], treatment of the PCR products with endonuclease V was expected to result in hydrolysis of the second phosphodiester bond 3′ to dI [51]. Removal of the weakly bound 4 bp ssDNA stretch creates cohesive ends compatible with those generated by the restriction enzyme KpnI. Figure 2 illustrates a similar case of cohesive end creation, whereas the design of the forward primer results in an overhang compatible with that of a different Type II restriction enzyme (see Figure 1 for all cohesive ends created in this study). It should be noted that the base-pairing properties of dI [43] will generate a sequence distribution at the corresponding position on the opposing strand of the PCR-generated dsDNA, which is discussed in the Conclusions section.

**Table 1:** Oligonucleotide sequences

**Oligonucleotide**	**Sequence (5′ - 3′)**
pUCamp_f	GT**I**CC TATGAGTAAACTTGGTCTGACAGTTACC
pUCamp_r	GT**I**CC GTCATCACCGAAACGCGCG
RFP-dev_f	TG**I**AG GCGCAACGCAATTAATGTGAG
RFP-dev_r	AC**I**TC GTTATTAAGCACCGGTGGAGTG
MITF_f	AG**I**TC ATGCTGGAAATGCTAGAATACAG
MITF_r	GT**I**CCTCA ACACGCATGCTCCGTTTCTTC
Mitf-FL_f	CATGCTAGCATGCTGGAAATGCTAGAATACAGTC
VR2_r	ATTACCGCCTTTGAGTGAGC

Deoxyinosine residues are shown in bold, primer add-on tails are underlined and separated from regions complementary to the target sequences by spaces.

With the two synthetic oligonucleotides, PCR was conducted using *Taq* DNA polymerase and a total of 27 amplification cycles. Subsequent to DNA purification, endonuclease V treatment and preparative agarose gel electrophoresis were performed. Ligation reactions were prepared with KpnI-digested and dephosphorylated pIRES2-EGFP or pSB1C3, respectively. Next, competent *E. coli* XL-1 Blue cells were transformed. After overnight incubation at 37°C, clones were found to grow on LB agar plates supplemented with ampicillin in addition to either kanamycin or chloramphenicol, respectively. Accordingly, the insert DNA was successfully integrated into the vector backbone and the amplified antibiotic resistance cassette (AmpR) was functional *in vivo*.

**Figure 3:**
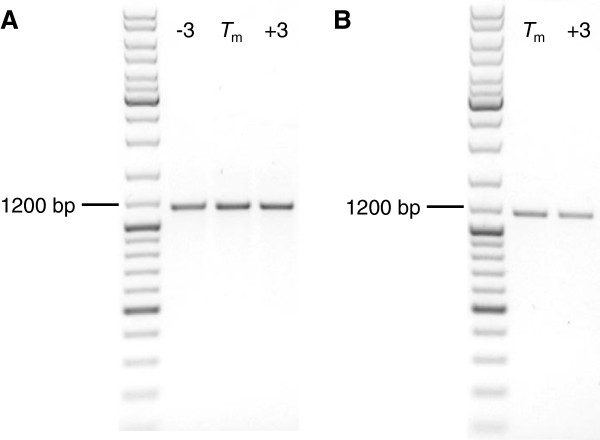
**Robust PCR-amplification of insert DNA fragments using deoxyinosine-containing primers.** Analytical agarose gel electrophoresis of PCR products produced by *Taq* polymerase using either plasmid DNA **(A)** or *E. coli* colonies **(B)** as template material. Relative to the calculated *T*_m_, annealing temperatures used for PCR cycling are indicated for each lane.

PCR was repeated with an annealing temperature gradient spanning *T*_m_ ± 3°C. As shown in Figure 3A, products of the expected size were formed in all cases with comparable quantities. Other, non-specific bands were not detected. Consequently, the presence of the dI-containing overhang did not hinder binding of the primers to the complementary plasmid DNA. This is consistent with earlier studies [52]. To test whether *E. coli* colonies can serve as a direct source for the target DNA, colony PCR was performed using XL-1 Blue cells transformed with the template plasmid. Cycling and reaction conditions were kept identical except for the initial denaturation, which was extended to 3 min to facilitate cell disruption and DNA release. Figure 3B shows that specific PCR products undistinguishable from those created by amplification from plasmid DNA were formed.

While simple co-transfection of vector and insert DNA fragments, each with large homologous regions (> 10 bp) at both ends, can create recombinant plasmids [13,14], we found no recombinant clones when the endonuclease V treatment of the insert DNA was omitted. Ligation reactions performed with only insert or vector DNA, respectively, also did not yield ampicillin-resistant clones.

### Directional cloning of a RFP reporter device

**Figure 4:**
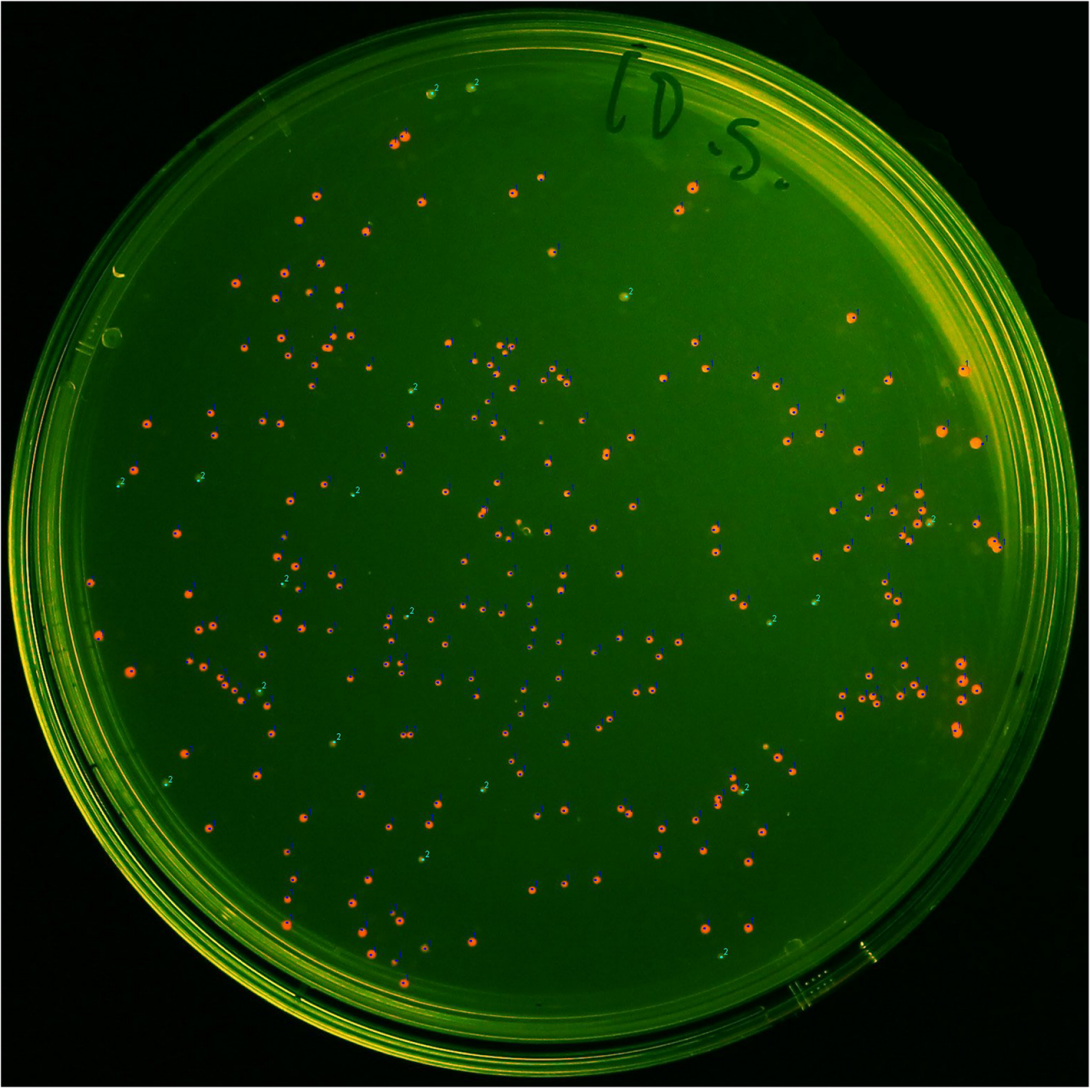
**mRFP1-positive clones obtained by cloning an RFP-coding device into pUC19.** Recombinant *E. coli* colonies expressing mRFP1. Detection was facilitated via excitation at 505 nm. Manual counting yielded a positive fraction (dark blue markers) of about 92.4% (233 of 252 colonies), cyan markers present negative colonies with poor or no fluorescence.

Since functional selection for the insertion of the ampicillin resistance cassette into plasmid vectors (previous chapter) did not yield information about the background of erroneous, empty or incomplete ligation events, a screening method for positive clones was employed. The red fluorescent protein (RFP) coding device BioBrick BBa_J04450 was chosen since mRFP1 expression by *E. coli* is easily detected [53]. Primers RFP-dev_f and RFP-dev_r (Table 1) were used to amplify an 830 bp region which comprises the *E. coli* lactose (*lac*) operon promoter including CAP and RNA polymerase binding sites, a ribosome binding site and a coding region for mRFP1 followed by a double TAA stop codon. The *T*_m_ value of the primer-template duplexes was 53–55°C. A total of 31 cycles were used for *Taq* polymerase-based amplification. After treatment with 5 u of *Tma* endonuclease V, a ligation reaction with dephosphorylated pUC19 digested with AatII and PstI was set up using a molar insert to vector ratio of 7:1. Transformation of *E. coli* XL-1 Blue yielded 252 colonies, of which 233 were classified as RFP-positive by manual counting (see Figure 4). Five apparently RFP-negative clones were used to inoculate 5 mL LB medium containing 100 μg/mL ampicillin. After overnight incubation at 37°C and 200 rpm, the liquid cultures possessed no or only slightly red color, respectively. DNA sequencing revealed that all clones carried an RFP coding device insert. Evidently, the observed low or missing red fluorescence was caused by point mutations or single base pair deletions in the mRFP1 coding sequence. Given the high number of PCR cycles used to produce the insert DNA fragment, this outcome was not unexpected. Assuming a constant amplification fidelity, the range of reported *Taq* polymerase error rates of 8 × 10^-6^ - 2 × 10^-4 ^[54,55] corresponds to a fraction of 25.7 - 100% PCR products with one or more base substitutions. In addition, plasmid DNA from seven fluorescent clones was sequenced. One clone carried two mutations of which one was silent; another clone carried one silent mutation. Five clones were free of mutations in the mRFP1 coding region. This corresponds to a total error rate of about 6 × 10^-4^, which fits the expected range (see above). Based on the frequency of RFP-positive clones, the efficiency of RFP-device insertion into the plasmid vector was ≥ 92.4%.

### Directional cloning of an eukaryotic coding sequence

To further explore the capabilities and limits of the cloning method, we chose to amplify the coding region of the *Mus musculus* microphthalmia-associated transcription factor (Mitf). The oligonucleotides MITF_f and MITF_r (Table 1) were designed for the amplification of a 1270 bp DNA fragment. The regions complementary to the template molecule were 24 or 21 bp in length, respectively, and a TGA stop codon was introduced via an overhang in the reverse primer. The oligonucleotide design was set up for the generation of cohesive ends corresponding to those created by the restriction enzymes SacI and KpnI as shown in Figure 2. It should be noted that with two internal SacI and one internal KpnI recognition sites, this DNA fragment cannot be cloned accordingly into the multiple cloning site of the vector using the conventional restriction-ligation strategy. The cycle number for *Taq*-based PCR was decreased to 19 in order to reduce the reaction time and the frequency of PCR errors. Following treatment with 5 u *E. coli* endonuclease V, purified insert DNA fragments were used in five- or 10-fold molar ratios relative to digested and dephosphorylated pBluescript II KS(+). Competent *E. coli* XL-1 Blue and BL21 strains were used for transformation of the ligation reactions. The five-fold molar excess of insert DNA fragments yielded 137 or 444 colonies, respectively, while the 10-fold excess yielded 83 or 456 colonies, respectively. Consequently, no profound differences were observed from the two different ratios of insert to vector molecules used in the individual ligation reactions. In order to detect the fraction of clones carrying the Mitf target DNA fragment inserted into pBluescript II KS(+) in correct orientation, a colony PCR assay was performed with colonies of both strains. Oligonucleotides Mitf-FL_f and VR2_r (Table 1) were used, with the first being complementary to the insert DNA sequence and the second to the vector backbone in reverse direction relative to the expected insert orientation. All 19 colonies tested were positive (data not shown), indicating the presence of the Mitf coding sequence inside the target plasmid in correct orientation. DNA sequencing of five additional randomly picked clones was performed, each using flanking forward and reverse primers. One clone showed a large plasmid backbone deletion of approximately 1.9 kb. The remaining four clones carried correct junction sites and the expected insert. Despite the relatively large amplicon size for a *Taq*-based PCR, two clones had full-length inserts free of mutations. The cloned Mitf coding sequence of the other two clones had two or four mutations, respectively.

### High fidelity cloning

Although robust, primer extension reactions using *Taq* DNA polymerase suffer from relatively low fidelity of the enzyme, restricting the cloning of DNA fragments to a maximum length of about 1.5 kb. As shown by Eckert and Kunkel, improvements in fidelity can be reached by optimization of the PCR conditions [54].

Cloning of even larger DNA fragments demands the use of a DNA polymerase with proofreading capability, thus 3′-5′ exonuclease activity. Several polymerases of archeal origin were reported to be unable to amplify deaminated nucleotides efficiently [56]. We found the enzymes Q5 (formulated with or without an aptamer-based inhibitor for hot start functionality), Phusion, *PfuUltra* II and Deep Vent_R_ failing to amplify DNA fragments when dI-containing oligonucleotide primers were used (data not shown).

However, with *PfuTurbo C *_*x*_ Hotstart, one exception was found. This *Pfu* DNA polymerase mutant was engineered to overcome uracil stalling. According to the manufacturer’s description, this enzyme possesses a fidelity equivalent to that of the wild-type protein and allows generation of PCR products exceeding a length of 6 kb [57]. While all other tested proofreading enzymes failed to generate PCR products suitable for endonuclease V-mediated cloning, all three types of recombinants (pIRES2-AmpR, pUC19-mRFP1, pBSK-Mitf) were successfully created by using the *Pfu* DNA polymerase mutant in place of the *Taq* enzyme. Without PCR optimization, comparable numbers of colonies were obtained using a molar vector to insert ratio of 1:8 (as summarized in Table 2). Ligation reactions using KpnI-linearized pIRES2-EGFP and PCR products treated with *E. coli* EndoV yielded 384 ampicillin-resistant colonies. Expression of mRFP1 was detected in 97 out of 113 clones, equivalent to a cloning efficiency of 85.8%. An increased molar vector to insert ratio of 1:10 yielded 170 colonies, of which 153 (90%) were fluorescent. Significantly lower in number, the origin of the 26 positive clones generated using a ratio of 1:8 without EndoV treatment remains unknown. Presumably, *in vivo* recombination events occurred.

**Table 2:** Cloning efficiencies using a proofreading DNA polymerase

**Ligation reaction**	**EndoV**	**Number of colonies**	**Fraction positive**
pBSK + Mitf PCR product	+	997	97.5% (39/40)
pBSK + Mitf PCR product	-	5	ND
Mitf PCR product	+	0	
pBSK		10	ND
pIRES2-EGFP + AmpR PCR product	+	384	100%
pIRES2-EGFP		0	
pUC19 + mRFP1 PCR product	+	113	85.8%
pUC19 + mRFP1 PCR product	-	26	100%
mRFP1 PCR product	+	0	
pUC19		0	

Ligation reactions were set up using plasmid vector DNA linearized by restriction endonuclease treatment. Column two indicates whether the PCR-generated insert was treated with endonuclease V. pBSK denotes pBluescript II KS(+). Fractions of positive clones were determined by red fluorescence (mRFP1 PCR product), growth in the presence of ampicillin (AmpR PCR product) or colony PCR (Mitf PCR product, 40 clones tested), respectively. ND = not determined.

**Figure 5:**
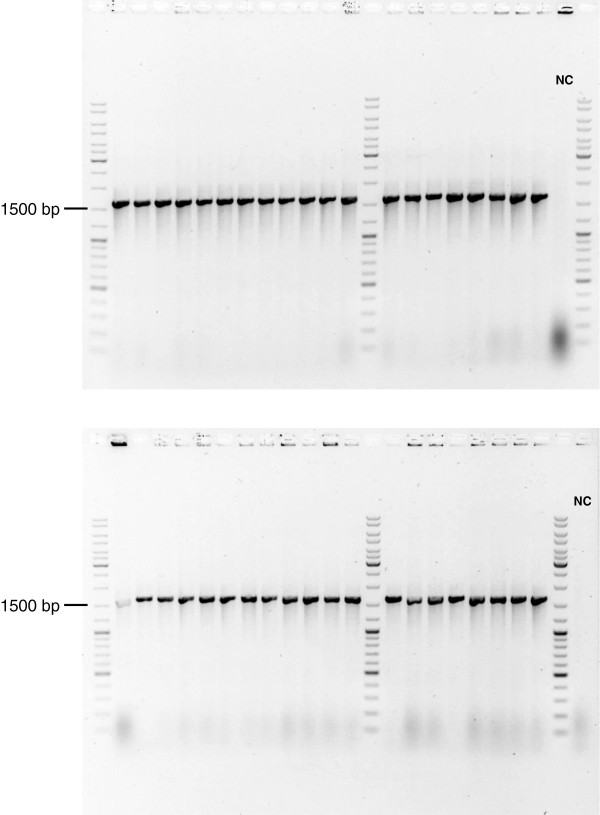
**Colony PCR screening to detect successful cloning of Mitf.** Presence of the Mitf coding region inside the plasmid vector pBluescript II KS(+) in correct orientation detected by colony PCR and analytical gel electrophoresis. From 40 individually tested colonies, 39 were judged positive as evident from the amplification of a DNA fragment (expected size: 1621 bp). Colonies from the same *E. coli* strain transformed with plasmid DNA lacking the Mitf coding region served as negative control (NC).

The Mitf PCR product was successfully cloned into the multiple cloning site of plasmid vector pBluescript II KS(+). Transformation of ligation reactions containing only the linearized plasmid vector yielded five colonies while the addition of the EndoV-treated PCR products resulted in 997 cfu. With 816 cfu, increasing the molar insert to vector fragment ratio to 10:1 did not result in a higher number of transformants. Without EndoV treatment, transformation of the corresponding ligation reaction resulted in only 10 colonies, demonstrating that deoxyinosine 3′ endonuclease activity is a strict requirement for the cloning strategy to work. Further analysis of the recombinants using colony PCR indicated that from 39 clones, 38 carried the Mitf coding sequence in correct orientation (Figure 5). This corresponds to a cloning efficiency of 97.5%.

**Figure 6:**
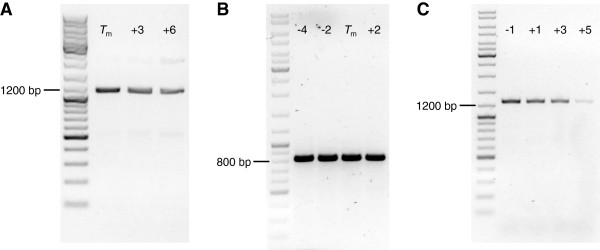
**Insert DNA fragment generation using a proofreading DNA polymerase.***PfuTurbo C*_*x*_ Hotstart polymerase was used for PCR amplification of insert DNA fragments using two deoxyinosine-containing oligonucleotides. Analytical agarose gel electrophoresis was performed with PCR products comprising the ampicillin resistance cassette **(**1114 bp, **A)**, the mRFP1 reporter device **(**830 bp, **B)**, and the Mitf coding sequence **(**1270 bp, **C)**. Annealing temperatures which were used for PCR cycling are indicated relative to *T*_m_ for each lane.

To our knowledge, this study is the first to demonstrate that a *Pfu* DNA polymerase mutant can achieve exponential DNA amplification in PCR using two deoxyinosine-containing oligonucleotides. Gill *et al.* have reported that the mutant enzyme V93Q can extend duplexes with modified primers, while exponential amplification fails when dGTP is replaced by dITP [58]. Using dI-containing oligonucleotides, primer extension reactions with the wild-type enzyme were reported to fail [44,59]. Using *PfuUltra* II Fusion HS, we indeed observed no exponential amplification (data not shown). Relative to *Taq* DNA polymerase, the mutant *Pfu* enzyme proved more sensitive towards high annealing temperatures. The target PCR product yield was found to be optimal when the primer annealing steps were performed at *T*_m_ - 3°C and decreased as soon as *T*_anneal_ exceeded *T*_m_ (compare Figure 6). Only in one case, nonspecific by-products were observed, namely for the ampicillin resistance cassette PCR conducted with *T*_anneal_ ≥ *T*_m_ + 3°C.

## Conclusions

The developed method allows the creation of PCR fragments carrying cohesive ends compatible to those of Type II restriction endonucleases which create 4 bp 3′ overhangs, as demonstrated herein for four different restriction enzyme recognition sites. To date, 21 enzymes of this type are commercially available [60]. The key advantage of our approach is the independence of the insert DNA sequence, which can - in contrast to the conventional cloning method - internally carry the recognition sequences of the restriction enzymes used for the digestion of the target plasmid vector. Consequently, only the approximate insert length (for PCR extension time determination) and its terminal sequences (for primer design) must be known. The developed method is straightforward and requires only minimal amounts of template DNA, e.g. 1 ng plasmid DNA or a single *E. coli* colony, for insert generation. With sufficient amounts of target plasmid vector at hand, all cloning steps can be performed in a single day finished by overnight incubation of transformed *E. coli* cells.

In contrast to other cloning strategies (e.g. In-Fusion cloning) [37], cohesive terminal sequences are created via primer overhangs only four nucleotides in length. As a result, the PCR-primers used for insert DNA generation remain short, minimizing the chance for secondary structure and primer-dimer formation as well as synthesis errors to occur. Including a single deoxyinosine residue, this type of modification is cost-efficient and available from commercial suppliers. Even at small synthesis scale, shipped primer amounts are good for several hundred PCRs. Therefore, the method is particularly cost-effective when primers can be reused, e.g. for cloning of individual mutants in the context of libraries. By employing two different overhangs, no multiple insertions were observed and cloning was directional as anticipated. Consequently, the developed method is most suitable to use when no appropriate pair of Type II restriction endonucleases for the conventional restriction-ligation strategy is at hand or available. As a result, a reduced number of such enzymes has to be maintained in the laboratory. The new strategy is also particularly suited when ligation-independent cloning (LIC) methods and techniques based on homologous recombination fail, for instance when the required homologous regions cannot be created via PCR. Although in principle possible, we recommended to avoid PCR-amplification of the vector DNA, as it is prone to introduce PCR errors. For the same reason, cloned insert sequences should be verified by sequencing.

Assuming an equal incorporation probability for all four canonical nucleotides when the DNA polymerase encounters the dI residue on the template strand, one out of four created insert termini can be ligated to a cohesive vector DNA end (compare Figure 2). It is thus conceivable that the ligation efficiency benefits from even higher molar insert to vector ratios. Based on our experiences, however, ratios between 5:1 and 8:1 are optimal. It should be emphasized that for all plasmid clones described within this study, the created ligation sites presented an exact match to the overhang of the vector fragment. Evidently, only PCR amplicons having precisely matching cohesive ends hybridize efficiently with vector molecules. Consequently, inserts which contained mismatches at the position complementary to the dI residue were not ligated to the linearized vector at detectable frequencies. Note that ligation conditions were chosen according to the manufacturer’s recommendation for T4 DNA ligase and cohesive ends. Conditions potentially favoring the formation of wrong pairings (e.g. low temperatures) should be avoided. While sufficient colony numbers were obtained in all cases, it should be noted that competent cells with relatively low transformation efficiency were used intentionally.

Although the terminal transferase activity of *Taq* DNA polymerase [61] could potentially cause ligation problems, additional nucleotides flanking the ligation sites or mutations therein were never observed. Inexpensive and robust, this enzyme is recommended for cloning of insert sequences up to 500 bp in length. Compatibility with the *PfuTurbo C*_*x*_ Hotstart DNA polymerase relieves the limitations generated by the relatively low fidelity of the *Taq* enzyme and greatly expands the application range of the cloning method. It is further possible that the absence of terminal transferase activity leads to higher cloning efficiencies compared to *Taq* polymerase.

With its broad buffer compatibility, *E. coli* endonuclease V allows its combined use with more than 200 commercially available restriction enzymes [9]. This could further expand the range of cloning options by generating one cohesive end via EndoV and one via a Type II restriction endonuclease. In addition, this enzyme can be heat-inactivated and requires an incubation temperature of 37°C in contrast to the highly stable *Thermotoga maritima* enzyme. Since a fill-in reaction by the DNA polymerase cannot take place, a direct addition of endonuclease V to the PCR mixture after thermocycling is also conceivable.

## Methods

### Plasmids and strains

Target plasmid vectors suitable for propagation in *E. coli* were pUC18 and pUC19 [62], pIRES2-EGFP (Clontech, Mountain View, CA, USA) and pBluescript II KS(+) [GenBank: X52327.1]. pSB1C3 and the RFP coding device BBa_J04450 were obtained from the Registry of Standard Biological Parts [63]. pAR200d-Mitf_FL is a derivative of pQE16 (Qiagen, Hilden, Germany) containing the coding sequence of the *Mus musculus* microphthalmia-associated transcription factor (Mitf)[GenBank: Z23066.1]. Competent *Escherichia coli* XL-1 Blue (Stratagene; now Agilent Technologies, Böblingen, Germany) and BL21-cells (Novagen, Darmstadt, Germany) were prepared by standard CaCl_2_ protocol. Transformation efficiencies determined as cfu per μg pUC18 plasmid DNA reached 1–3 × 10^6^ for XL-1 Blue and 3–4 × 10^6^ for BL21.

### Design of deoxyinosine-containing primers

All oligonucleotides used in this study were obtained from Sigma-Aldrich (Taufkirchen, Germany) in desalted quality without further purification. Primers were dissolved in water and stored at -20°C. All *T*_m_ values reported in this study correspond to theoretical values determined for complementary regions by the nearest-neighbor method using OligoCalc [64] with default parameters. In order to create 3′ protruding ends by endonuclease V treatment of the PCR products, a single deoxyinosine residue was placed at the third position of the primer 5′ end (compare Figure 1). DNA segments complementary to the ends of the template molecule were designed to reach *T*_m_ values of ≥ 53°C.

### PCR-based amplification of target DNA fragments

*Taq* DNA polymerase and dNTP mix were obtained from New England Biolabs (Frankfurt am Main, Germany). The supplied Standard *Taq* Reaction Buffer containing 1.5 mM MgCl_2_ was used. Reactions with a total volume of 50 μL further contained 50 μM of each dNTP, 0.2 μM each primer, 1 ng template DNA and 2.5 u enzyme. Thermocycling was performed using a Mastercycler gradient (Eppendorf, Hamburg, Germany) with a heated lid and the following common parameters: initial denaturation 95°C 30 s; amplification (95°C 25 s, *T*_anneal_ (as calculated) 25 s, 68°C 60 s per kb) × 19–31 cycles; final extension 68°C 3 min. Unless stated otherwise, *T*_anneal_ equals to the calculated *T*_m_ value minus 3°C. For colony PCR, small samples of *E. coli* colonies served as the template DNA source. *PfuTurbo C*_*x*_ Hotstart DNA polymerase (Agilent Technologies, Böblingen, Germany) was used for high fidelity PCR. Reactions contained the supplied buffer and final concentrations of 50 μM each dNTP, 0.2 μM each primer, 1 ng template DNA and 2.5 u of enzyme. Thermocycling was performed using the following parameters: 95°C 2 min; (95°C 20 s, *T*_anneal_ 20 s, 72°C 60 s per kb) × 25 cycles; 72°C 3 min. *PfuUltra* II Fusion HS DNA polymerase was purchased from Agilent Technologies (Böblingen, Germany). The DNA polymerases Q5 High-Fidelity, Q5 Hot Start High-Fidelity, Deep Vent_R_ and Phusion High-Fidelity were purchased from New England Biolabs (Frankfurt am Main, Germany). DNA concentrations were determined using a NanoDrop 2000 micro-volume UV-Vis spectrophotometer (Thermo Fisher Scientific, Schwerte, Germany). Agarose gels for PCR product analysis or purification, respectively, were prepared using Agarose Standard (Carl Roth GmbH, Karlsruhe, Germany) and TAE buffer. GeneRuler DNA ladder mix (Thermo Fisher Scientific, St. Leon-Rot, Germany) was used as a size marker. DNA was stained using GelRed (Biotium Inc., Hayward, CA, USA). PCR products were visualized under UV transillumination. Pictures were taken using an EOS 1100D Digital SLR camera (Canon, Krefeld, Germany) equipped with a Hoya K2 HMC filter (Hapa-Team, Eching, Germany). In order to facilitate the visual detection of faint bands, adjustments in greyscale levels were performed on the entire digital image. Silica membrane-based PCR product purification was performed using a NucleoSpin Extract II kit (Machery-Nagel, Düren, Germany).

### Endonuclease V treatment

*Escherichia coli* or *Thermotoga maritima* endonuclease V (EndoV) were obtained from New England Biolabs (Frankfurt am Main, Germany) or Thermo Fisher Scientific (St. Leon-Rot, Germany), respectively. DNA treatments were performed in the supplied buffers for 45 min at 37°C or 60°C, respectively.

### Ligation with plasmid vector fragments

Plasmid vector DNA fragments were produced by restriction endonuclease treatment. All restriction enzymes (EC 3.1.21.4) were of Type IIP [65] and were obtained from New England Biolabs (Frankfurt am Main, Germany), as High Fidelity (HF) versions if available. Reactions were performed at 37°C for at least 2 h using the supplied NEBuffer 4 and BSA solution. Vector DNA fragments were purified subsequent to agarose gel electrophoresis using a NucleoSpin Extract II kit (Machery-Nagel, Düren, Germany). Antarctic Phosphatase from New England Biolabs (Frankfurt am Main, Germany) was used to release the terminal 5′ phosphate groups; incubation and heat inactivation were performed as recommended by the manufacturer. Without further purification, the reaction products were used for ligation reactions. Different molar ratios of insert to vector DNA as well as 1 Weiss unit of T4 DNA Ligase and the supplied buffer from Thermo Fisher Scientific (St. Leon-Rot, Germany) were used. With a total volume of 10 μL, the reactions were incubated for 30 min at room temperature (20–25°C). A 2 μL sample was used for *E. coli* transformation (50 μL cell suspension) by heat shock.

### Analysis of transformants

The presence of a functional ampicillin resistance cassette (AmpR) was tested by transferring freshly transformed *E. coli* cell suspensions onto LB agar plates supplemented with 50 μg/mL ampicillin in addition to either 50 μg/mL kanamycin (pIRES2-EGFP vector backbone) or 25 μg/mL chloramphenicol (pSB1C3 vector backbone), respectively. Colony forming units (cfu) were determined as described [66] using ImageJ (National Institutes of Health, Bethesda, MD, USA). Expression of mRFP1 [53] from the RFP coding device was visible to unaided eyes under day light. Epi-illumination pictures of red fluorescent colonies were taken by using 505 nm cyan LEDs (Winger Electronics, Dessau-Roßlau, Germany) and an EOS 1100D Digital SLR camera (Canon, Krefeld, Germany) equipped with a UV-filter.

## Abbreviations

AmpR: Ampicillin resistance gene; dI: Deoxyinosine nucleotide; dU: Deoxyuracil nucleotide; EndoV: Endonuclease V; LIC: Ligation-independent cloning; Mitf: Microphthalmia-associated transcription factor; RFP: Red fluorescent protein mRFP1; Tm: Calculated midpoint of thermal dsDNA melting; Tanneal: PCR annealing temperature; Tma: *Thermotoga maritima*; UDG: Uracil DNA glycosylase

## Competing interests

The authors declare no competing interests.

## Authors’ contributions

KM and TB conceived the method. TB designed and performed research, analyzed data and wrote the manuscript. KM contributed to writing the manuscript. KMA provided scientific advice and the supporting infrastructure. All authors read and approved the final manuscript.
